# Experimental data on the characterization of hydroxyapatite produced from a novel mixture of biowastes

**DOI:** 10.1016/j.dib.2022.108305

**Published:** 2022-05-21

**Authors:** Obinna Anayo Osuchukwu, Abdu Salihi, Ibrahim Abdullahi, David Olubiyi Obada

**Affiliations:** a Department of Mechanical Engineering, Bayero University, Kano, Kano State 700241, Nigeria; b Department of Mechanical Engineering, Ahmadu Bello University, Zaria, Samaru, Zaria, Kaduna State 810212, Nigeria; c Africa Centre of Excellence on New Pedagogies in Engineering Education, Ahmadu Bello University, Zaria, Samaru, Zaria, Kaduna State 810212, Nigeria; d Multifunctional Materials Laboratory, Shell Office in Mechanical Engineering, Ahmadu Bello University, Zaria, Samaru Zaria, Kaduna State 810212, Nigeria

**Keywords:** pH adaptability, Absorbed water, Mechanical strength, Microstructure, Sol-gel

## Abstract

The purpose of this data narrative is to report the morphological structures, functional groups, elemental composition, pH adaptability and mechanical properties of hydroxyapatite (HAp) biomaterials synthesized from a novel mixture of biowastes (bovine and catfish bones) by a simple sol-gel method assisted with sintering at 900 °C. The produced powders were homogenously mixed by the sol-gel approach at different weights (depicted by sample nomenclature) and characterized using scanning electron microscopy (SEM) equipped with electron dispersive X-ray analysis (EDX), X-ray fluorescence (XRF), Fourier Transform Infrared Spectroscopy (FT-IR), immersion in phosphate buffer saline (PBS), and mechanical measurements (hardness and fracture toughness). The SEM micrographs revealed pore interconnections in all samples. The EDX analysis revealed that the as-sintered HAp samples had Ca/P weight ratios of 2.38, 2.51, 2.86, 2.89, and 3.10 for C100, BC 75/25, BC 50/50, BC 25/75, and B100 samples, respectively. The FT-IR spectra was typical of the bands associated with hydroxyapatite (i.e., those associated with the PO_4_^3−^ , CO_3_^2-^ groups and absorbed water). The prepared biomaterials showed pH adaptability and good mechanical strength.

**Specifications Table**

**Table:** 

Subject	Engineering
Specific subject area	Biomedical Materials: Synthesis and Characterization
Type of data	Table, Image, Chart, Graph, Figure
How the data were acquired	SEM/EDX, FT-IR, XRF, Immersion in Phosphate Buffer Saline, Mechanical Measurements. The microstructure of the samples was studied using a Phenom ProX Desktop Scanning Electron Microscope (SEM) equipped with EDX for elemental mapping and operated at 15 kV. Each sample was examined under low magnification of 1000 x. The EDX maps revealed the weight percentages of each element in the bulk of the synthesized HAp.Surface chemistry data was gathered using a Fourier Transform Infrared Spectrometer with a four-wavenumber resolution that operated between 4000 and 650 cm^−1^.
Data format	Raw, Analysed
Description of data collection	The data presented represents the morphological features, elemental composition, functional groups, pH adaptability profiles and mechanical measurement data of the synthesized biomaterials.
Data source location	• Institution: Bayero University and Ahmadu Bello University • City/Town/Region: Kano/Kano; Zaria/Kaduna • Country: Nigeria
Data accessibility	With the article & https://data.4tu.nl/account/home#/data

**Value of the Data**

• The data can be used to track changes in the characteristics of biomaterials made from a mixture of biowastes and can be useful to researchers working on biowaste valorization.
• The SEM images in this article provides data that reveals the interconnectivity of the pores inherent in the biomaterials.
• The elemental composition of the biomaterials reveals all the oxides present in the bulk of the mixture
• The FT-IR data reported in this article reveals the most prominent chemical groups in the FT-IR spectrum of HAp, such as OH^−^, $PO_{4}^{3-},\;CO_{2}^{3-}\;,$ OH^−^.
• The pH adaptability profiles and mechanical measurement data points towards the application potentials of the biomaterials
• Future synthesis of the novel biowaste mixtures in this study can benefit from the data highligted in this article.

## 1 Data Description

**Fig. 1:**
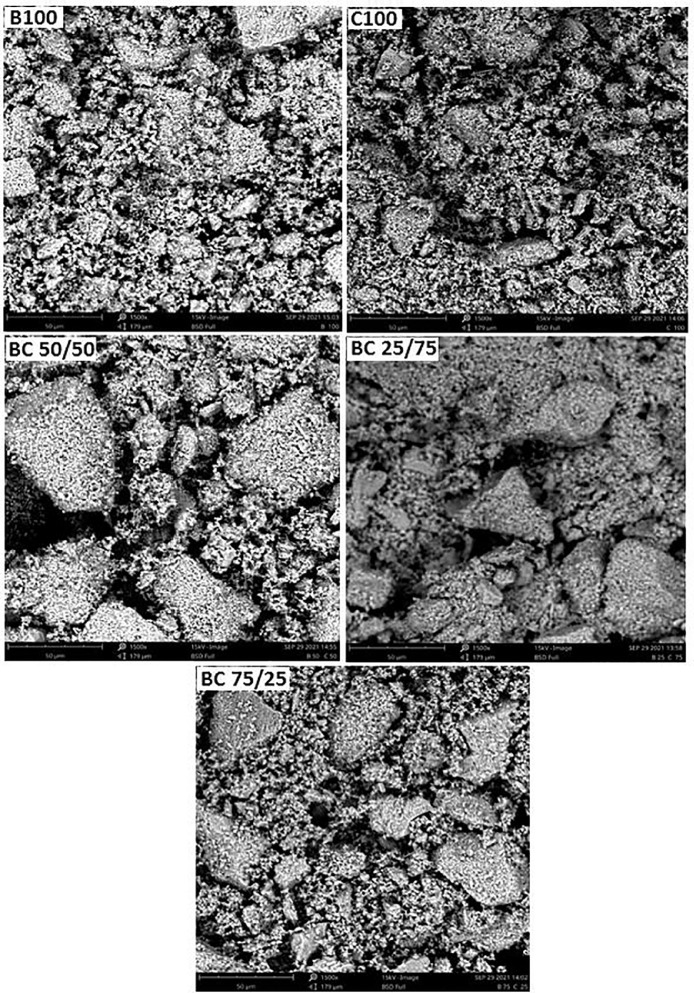
SEM micrographs of the HAp samples.

**Fig. 2:**
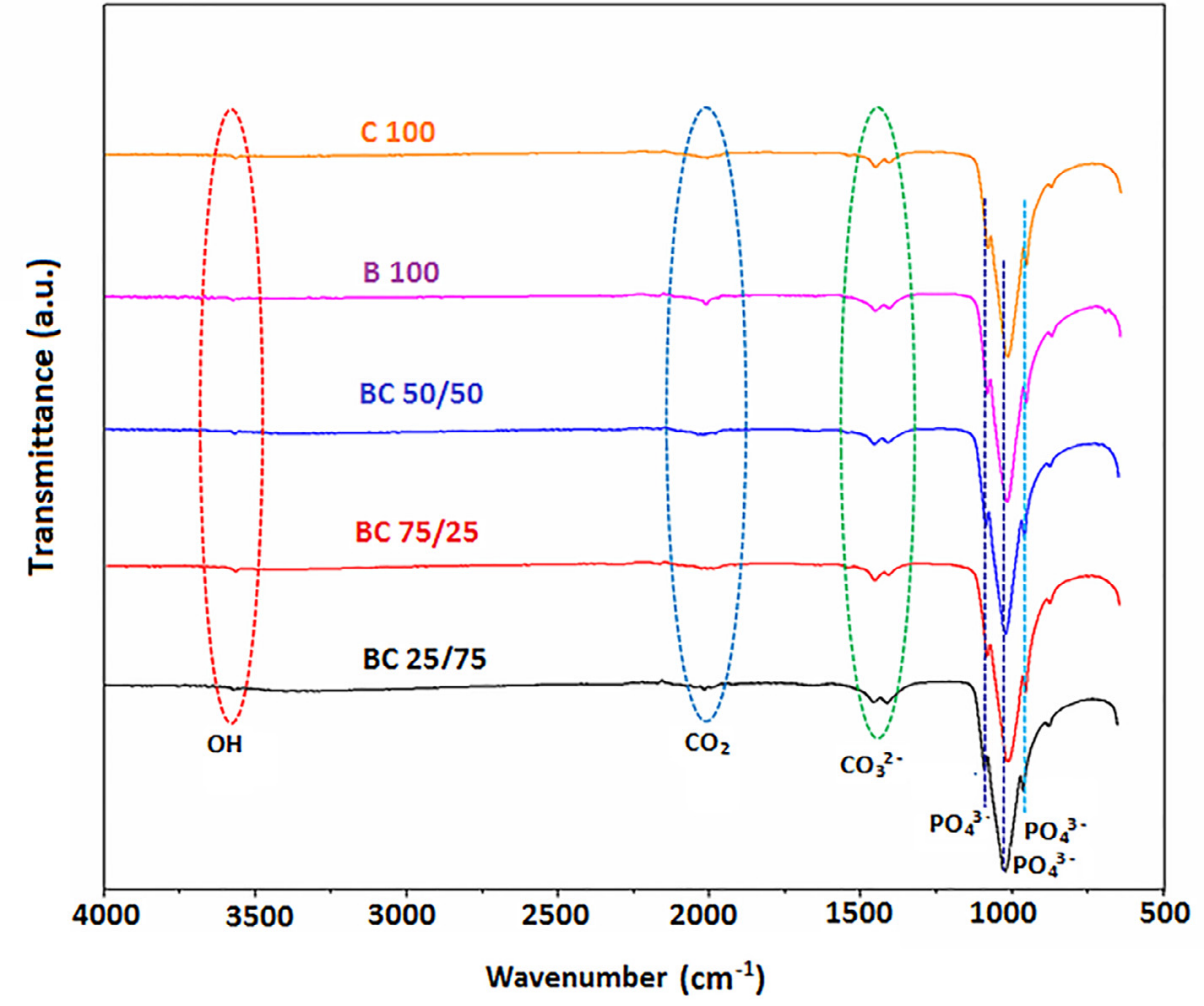
FT-IR spectrogram of the HAp samples.

**Fig. 3:**
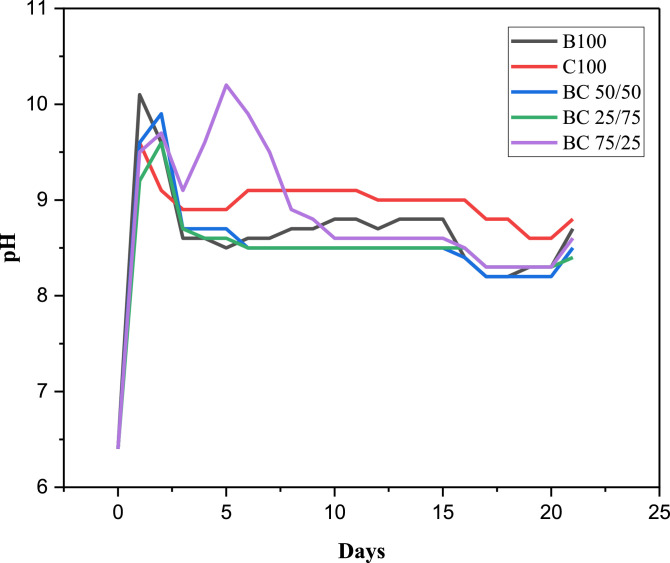
pH profiles of the HAp samples.

**Fig. 4:**
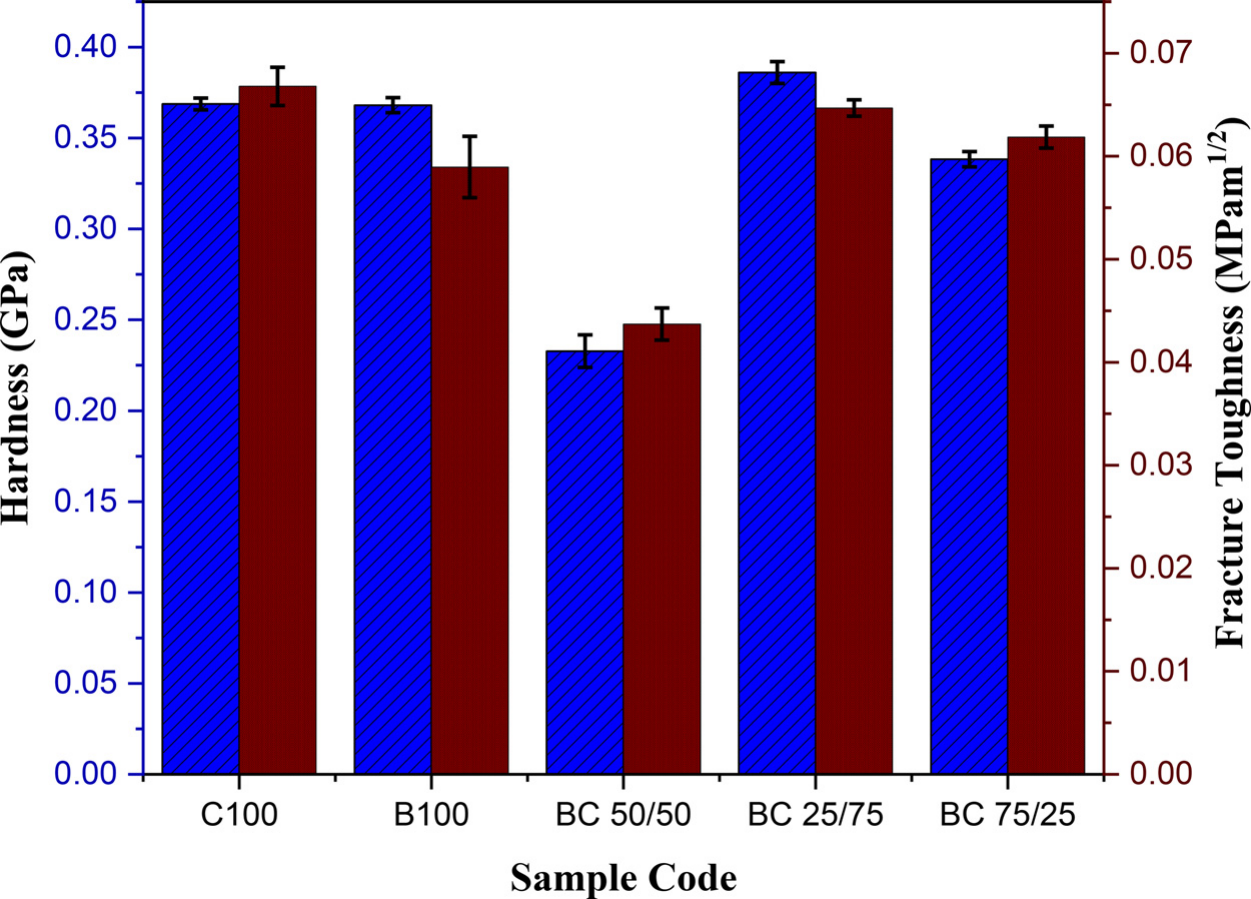
Mechanical measurement data for the HAp samples.

The dataset available for SEM, XRF, FTIR analyses, in addition to pH adaptability and mechanical measurements as shown in Fig. 1, Fig. 2, Fig. 3, Fig. 4 and Table 1, Table 2, Table 3, Table 4, are for the processed hydroxyapatite powders sintered at 900 °C. Fig. 1 shows the morphology of the samples at magnifications of 1000 x. Fig. 2 shows the FT-IR data (raw data of the spectrogram is presented as supplementary files). Fig. 3 shows the pH adaptability profiles, while Fig. 4 show mechanical measurement data. The elemental composition using XRF, elemental composition using EDX, Ca/P ratio in weight percentage, and FT-IR summary tables (Tables 1–4), show elemental oxides in the bulk of the samples, elemental composition of the samples, Ca/P ratios and obtained functional groups.

**Table 1:** (a) Elemental oxide composition of the HAp samples.

Concentration (wt.%)					
Compounds	B_100_	C_100_	B_75_C_25_	B_50_C_50_	B_25_C_75_
SiO_2_	0.633	1.09	0.683	1.891	1.515
V_2_O_5_	0.045	0.026	0.022	0.04	0.04
Cr_2_O_3_	0	0.004	0	0	0
MnO	0.083	0.065	0.052	0.069	0.071
Fe_2_O_3_	0.411	0.319	0.238	0.447	0.42
Co_3_O_4_	0.021	0.014	0.011	0.016	0.014
NiO	0.01	0.006	0.002	0.007	0.008
CuO	0.049	0.038	0.035	0.038	0.04
Nb_2_O_3_	0.006	0.004	0.004	0.005	0.007
MoO_3_	0.005	0.003	0.003	0.007	0.005
WO_3_	0.011	0.009	0.007	0.01	0.002
P_2_O_5_	26.737	32.264	31.652	27.846	28.279
SO_3_	0.098	0.433	0.289	0.197	0.326
CaO	66.294	61.383	63.568	63.667	65.363
MgO	2.55	0.396	0.198	1.913	0
K_2_O	0.071	0.142	0.086	0.089	0.107
BaO	0.19	0.05	0.135	0.071	0.046
Al_2_O_3_	1.443	1.792	1.532	2.114	2.031
Ta_2_O_5_	0.025	0.013	0.013	0.017	0.013
TiO_2_	0.108	0.003	0	0.029	0.033
ZnO	0.023	0.017	0.013	0.017	0.02
Ag_2_O	0.011	0.016	0.006	0.007	0
Cl	0.741	1.403	1.023	1.102	1.262
ZrO_2_	0	0.004	0	0.001	0.004
SnO_2_	0.307	0.373	0.314	0.26	0.248
SrO	0.128	0.132	0.115	0.14	0.145
**Sum**	**100**	**99.999**	**100.001**	**100**	**99.999**

The temperature selected for the synthesis of the mixture of biowastes in this study was 900 °C because it has been shown that at lower temperatures, there is a high level of reactivity of these powders [[1], [2], [3], [4], [5], [6], [7],^24^]. The SEM images as shown in Fig. 1 corresponds to the morphological features of the samples at 1000 x magnification. The grain distribution can be observed from these images. For all the samples, the grains exhibit several sizes and shapes with B100 and C100 samples showing more homogeneity in terms of the samples’ sizes and shapes. For the other samples (BC 50/50, BC 75/25 and BC 25/75), it can be observed that the big grains are composed of small particles agglomerated between them. A close look at the SEM images reveals denser microstructures for B100 and C100 samples, with these images showing finer particles with spaces between the particles assumed to be pores/voids. It is assumed that porosities are embedded between these large grains. The oval, spherical, and rod-like shapes evident in the samples can be attributed to carbonate, and hydroxyapatite. The architecture of the pores and relative densification observed from the images have an influence on the characteristics of the samples [[3], [4], [5],[16], [17], [18], [19], [20], [21], [22], [23]]. The XRF analysis carried out on all the five samples as shown in Table 1 reveal the characteristic concentration of typical oxides of CaO, P_2_O_5_, ZnO, and MgO etc. Particularly, the presence of MgO is important for bone metabolism and the development of artificial bones [13]. In this regard, the B100 sample shows more promise as compared to the other samples because it contains 2.55 wt% of magnesium oxide. EDX analysis was conducted to identify the compositional make-up of the samples. We were also interested in some additional elemental impurities which could be localized in the bulk of the samples and enhance their potentials for biomedical applications. The results in Table 2 show that the EDX results are consistent with those reported elsewhere [14]. Calcium (Ca), phosphorous (P), and oxygen (O) made up the majority of the samples, with minor elements like magnesium (Mg), strontium (Sr), and potassium (K) in good measure. The presence of these elements can enhance and expedite bone growth and new bone development in vitro and in vivo. Particularly, the presence of Mg is important for bone metabolism and the development of artificial bones [13]. In this regard, the B100 sample shows relative potentials as compared to the other samples in terms of its compositional make-up. From the EDX analysis, the calcium (Ca) and phosphorus (P) atoms present in weight percentages provides the mean relative calcium to phosphate ratios as shown in Table 3. From the data (Table 3), calculated Ca/P ratios for the samples B100, C100, BC 50/50, BC 75/25, and BC 25/75 were 3.10, 2.38, 2.86, 2.51, and 2.89, respectively. These values are different from the stoichiometric Ca/P ratio of hydroxyapatite (1.67). One possibility linked to this difference is the inclusion of a foreign crystal which are calcium rich compounds like CaO, Ca (OH)_2_ and CaCO_3_ or a possible mixture of the three. It was not possible to differentiate between these compounds because the identification of elements such as carbon was not possible with the method used. A high Ca/P ratio has been suggested to ensure optimal biocompatibility and chemical stability of the HAp in the implanted location when used as bone remodelling materials [15].

Fig. 2 depicts the Fourier Transform Infrared (FT-IR) spectrograph of the produced HAp samples along with a summary report in Table 4. It can be noticed that the FT-IR spectrum of the powders are typical of hydroxyapatite. The appearance of a number of bands (small and obvious peaks) in the range of 3572 cm^−1^, 2072 cm^−1^, 2002 cm^−1^, 1480–1500 cm^−1^, 1045 cm^−1^, and 1021 cm^−1^ matched the peaks of the HAp reference sample spectrum. The appearance of these peaks in the FT-IR spectrum can be ascribed to the presence of ions such as phosphate (PO_4_^3−^), hydroxyl (OH), and carbonate (CO_3_^2−^). The samples showed small bands around 2010 cm^−1^, and it is possible to ascribe this to the release of CO_2_ during heat treatment.

The pH adaptability profiles for all the samples as shown in Fig. 3 reveals that the pH value of the phosphate buffer saline (PBS) solution gradually increased for all the hydroxyapatite samples with gradients in the pH values during the 21 days of incubation. There was a considerable increase of pH for the BC 50/50 sample which hovered close to the neutral pH levels of 7.4. Such gradients shown in pH for the samples can be as a result of the dissolution of alkaline ions from the samples which compensates for the acidity of the PBS medium. Samples with more pH regulation tendencies have the potential to reduce the inflammation of soft and hard tissues during implantation.

The mechanical properties of the samples are presented in Fig. 4. Micro-hardness of cortical bones is in the 0.3–0.6 GPa range [7]. From the data presented, the micro-hardness for all the samples except BC 50/50 is out of this range which could suggest lesser potentials for hard tissue engineering. The fracture toughness of the samples is also presented in Fig. 4. The low fracture toughness values for all the samples with the highest being 0.07 MPa•m^1/2^ for the C100 sample could be as a result of the low compaction pressure (500 Pa) used in pelletizing the samples and relatively low sintering temperature. Higher sintering temperature has proven to be beneficial for increased mechanical properties of pelletized ceramic pellets [16], [17], [18], [19], [20], [21], [22], [23].

**Table 2:** EDX- elemental composition of the HAp samples.

Concentration (%)					
Elements	B100	C100	BC75/25	BC50/50	BC25/75
O	36.423	37.814	37.535	37.041	36.839
Mg	1.538	0.239	0.12	1.154	0
Al	0.764	0.949	0.811	1.119	1.075
Si	0.296	0.51	0.319	0.884	0.708
P	11.669	14.081	13.814	12.153	12.342
S	0.039	0.174	0.116	0.079	0.131
Cl	0.741	1.403	1.023	1.102	1.262
K	0.059	0.118	0.071	0.073	0.089
Ca	47.381	43.871	45.433	45.503	46.716
Ti	0.065	0.002	0	0.017	0.02
V	0.025	0.015	0.012	0.022	0.022
Cr	0	0.003	0	0	0
Mn	0.064	0.05	0.04	0.054	0.055
Fe	0.288	0.223	0.167	0.313	0.294
Co	0.016	0.011	0.008	0.012	0.01
Ni	0.008	0.004	0.001	0.005	0.006
Cu	0.039	0.03	0.028	0.03	0.032
Zn	0.019	0.014	0.011	0.013	0.016
Sr	0.108	0.111	0.097	0.118	0.122
Zr	0	0.003	0	0.001	0.003
Nb	0.005	0.003	0.003	0.004	0.006
Mo	0.003	0.002	0.002	0.005	0.004
Ag	0.01	0.015	0.005	0.006	0
Sn	0.242	0.294	0.247	0.205	0.195
Ba	0.17	0.045	0.121	0.064	0.041
Ta	0.02	0.011	0.01	0.014	0.011
W	0.009	0.007	0.006	0.008	0.001

**Table 3:** Calcium to phosphate ratio of the hydroxyapatite samples.

Sample Notation	B_100_	C_100_	B_50_C_50_	B_75_C_25_	B_25_C_75_
Calcium, Ca (wt%)	47.381	43.871	45.503	45.433	46.716
Phosphate, P (wt%)	11.669	14.081	12.153	13.814	12.342
Ca/P (wt%)	3.10	2.38	2.86	2.51	2.89

**Table 4:** Wavenumbers, chemical groups and description of the FT-IR spectrum of the HAp samples.

Sample notation and wavenumber (cm^−1^)							
B_100_	C_100_	B_50_C_50_	B_75_C_25_	B_25_C_75_	Chemical group	Description	Refs.
817, 961	817, 961	817, 961	817, 961	817, 961	$PO_{4}^{3-}$	Bending mode	[9–12]
1039	1039	1039	1039	1039	$PO_{4}^{3-}$	Antisymmetric widening mode	
1415, 1459	1415, 1459	1415, 1459	1415, 1459	1415, 1459	$CO_{3}^{2-}$	Substitutes of phosphate ion (i.e CO_3_ substituting for PO_4_) HAp is formed	[8,[10], [11], [12]]
2027, 2039	2027, 2039	2027, 2039	2027, 2039	2027, 2039	Absence of water	Under influence of thermal treatment, absorption band becomes invisible	[1,[3], [4], [5]]

## 2 Experimental Design, Materials and Methods

### 2.1 Preparation of the samples

Animal bones (Bovine) were sourced from an abattoir in Zaria metropolis, Nigeria, while catfish bones were sourced from local restaurants from the same location. The bones were deproteinized in an in-house developed oven. Next, the deproteinized bones from the two sources were calcined separately/independently for 2 (two) hours at 900 °C in a muffle furnace for a holding time of 2 h allowed to cool in the furnace before being crushed and sieved through a 100 µm sieve. The produced powders from the two sources after thermal treatment were weighed and mixed with a spatula in different proportions totaling 100 g (scale-down measures were applied) as shown in Table 5. Next, the as-mixed powders were further homogenized using the sol-gel method. The powders were poured in 150 ml of distilled water and placed on a hot plate/magnetic stirrer for 2 h under stirring. Next, the powders were mixed to dry under a step wise increase in temperature. The resultant gel was dried in the oven at 60 °C before characterization.

**Table 5:** Composition of the samples and their nomenclature.

S/N	Calcined powder from Bovine Source (g)	Calcined powder from Catfish Source (g)	Nomenclature
1	100	-	B100
2		100	C100
3	75	25	BC 75/25
4	25	75	BC 25/75
5	50	50	BC 50/50

**Fig. 5:**
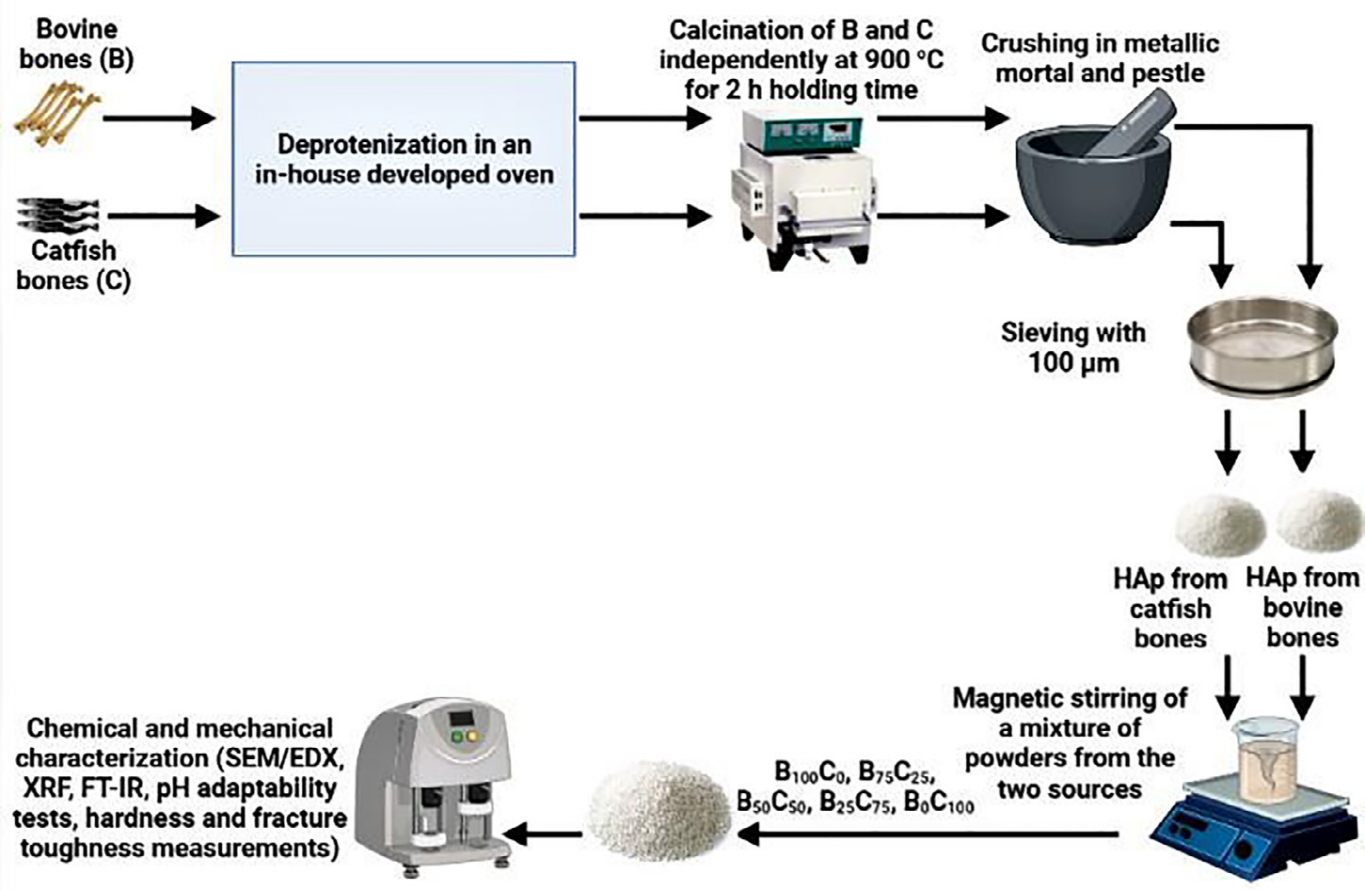
Schematic for hydroxyapatite preparation and characterization protocol.

The morphology of the synthesized samples was examined using scanning electron microscopy (SEM) (Phenom ProX Desktop equipped with EDX for elemental mapping and operated at 15 kV. Each sample was examined under magnification of 1000x. The EDX maps revealed the weight percentages of each element in the samples. XRF analysis was conducted using the XRS-FP analysis software for the elemental oxide composition of the hydroxyapatite samples. FT-IR spectrum of the samples was recorded in the 4000–650 cm^− 1^ range, collected by attenuated total reflectance (ATR-FTIR). For the pH adaptability experiments, the scaffolds were immersed in 5 ml of phosphate buffer saline (PBS, pH = 7.4) and placed in the incubator for 21 days at 37 °C. The pH of the solution was taken daily using a pH meter. For the mechanical measurements, low cold compaction pressure of 500 Pa was used to pelletize the samples. The microhardness of the samples was determined with Vickers’ microhardness tester. The obtained parameters from the hardness test were then used to calculate the fracture toughness, K_1c_ as described in [10]. Fig. 5 shows a schematic of the synthesis and characterization processes.

## Ethics Statements

Nil.

## CRediT Author Statement

**Obinna Anayo Osuchukwu:** Conceptualization, Methodology, Data curation, Investigation, Writing – original draft; **Abdu Salihi:** Supervision; **Abdullahi Ibrahim:** Reviewing and Supervision; **David Olubiyi Obada:** Conceptualization, Methodology, Data curation, Writing – original draft, Visualization, Investigation, Reviewing and Editing, Supervision.

## Funding Statement

The authors wish to acknowledge funding from Tertiary Education Trust Fund (TETFund), Nigeria under grant Ref: NRF_SETI_HSW_00714, 2020.

## Declaration of Competing Interest

The authors declare that they have no known competing financial interests or personal relationships that could have appeared to influence the work reported in this paper.

## Data Availability

FTIR data sheet, Mechanical Properties and Ph Adaptability Data for Hydroxyapatite Samples .xlsx (Original data) (https://data.4tu.nl/account/home#/data).

## Appendix Supplementary materials

Image, application 1

Image, application 2
